# Sagittal en bloc resection of primary tumors in the thoracic and lumbar spine: feasibility, safety and outcome

**DOI:** 10.1038/s41598-020-65326-0

**Published:** 2020-06-04

**Authors:** Lei Dang, Zhongjun Liu, Xiaoguang Liu, Liang Jiang, Miao Yu, Fengliang Wu, Feng Wei

**Affiliations:** Orthopaedic Department of Peking University Third Hospital, Beijing Key Laboratory of Spinal Disease Research, No 49. North Garden Road, HaiDian District, 100191 Beijing, China

**Keywords:** Spinal cord diseases, Surgical oncology, Surgical oncology

## Abstract

This study is to test feasibility, safety and the outcome of sagittal en bloc resection of paravertebral primary tumors in the thoracic and the lumbar spine. Sagittal en bloc resection was planned based on WBB classification and performed via combined anterior-posterior or anterior-posterior-lateral approach in 9 consecutive patients with aggressive benign or malignant paravertebral primary tumors in the thoracic and lumbar spine. Surgical margins were evaluated both radiologically and histopathologically. Follow-up data regarding survival rate, local control, morbidity, hardware failure and postoperative function were collected at around 2 years after surgery. En bloc resection was achieved in all patient with wide margin in 7/9 patients, marginal and intralesional margin in 2/9 patients. Survival rate and local control rate were 100%. There were 4/9 cases of major complications and 2/9 cases of minor complications with an overall morbidity rate of 67% (6/9). All but one patient with intraoperative spinal cord injury were free of neurological deficits and fully mobile in absence of any indication of hardware failure. With a careful choice of surgical procedure, sagittal en bloc resection of paravertebral primary tumor in the thoracic and lumbar spine is feasible, safe and effective.

## Introduction

Primary tumors of the spine are rare, accounting for 4–13% of all primary bone tumors^1^. Its estimated incidence is around 2.5–8.5 per 100,000 people per year^2^. Primary tumors can affect all regions of the spine; the thoracic and the lumbar spine are among the sites of predilection.

Enneking *et al*. proposed in 1980 a system that use clinical, imaging and histopathologic findings to stage biological behaviors of tumors of the bone and soft tissue. It was later applied to the spine^3^. For tumors at various stages, surgical planning must be based on what the procedure achieves in relation to the margin of the lesion, as the final pathological margin to be achieved is directly related to the prognosis. The most reliable surgical technique for removal of spine tumors is the en bloc resection technique, by which the tumor is removed as a single intact whole with a fully encased cuff of healthy tissue. During the past decade, total en bloc spondylectomy (TES) has become the gold standard technique for removal of spinal tumors for its high efficacy in local disease control and improved survival rate^4,5^. However, extensive dissection and removal of the entire vertebrae increases the risk of surgical complications such as vascular injury and nerve damage, loss of stability and perioperative mortality^6^. In 1996, Weinstein, Boriani and Biagini proposed the WBB staging system that classifies envelops of resection based on extension and location of the tumor^7^. It divides the vertebra into 12 radiating zones (zone 1 to 12 clockwise) and 5 layers (A to E from the prevertebral to the dural involvement) on the transverse plane, allowing a precise surgical resection to be planned in the closest safe zone to the margin of the tumor. This allowed them to propose the procedure of sagittal en bloc resection for tumors that grow eccentrically on one side of the vertebra(e) (within zone 2–5 or 7–11). In contrast to TES, this procedure is intended to remove the tumor with a safe margin while preserving the healthy part of the vertebra(e). By limiting dissection, it should reduce surgical complications and promote recovery. But given the rarity of primary spine tumors, there has been a lack of reports validating the feasibility and results of this technique.

This study is prompted to investigate the feasibility, complications and outcome of sagittal en bloc resection of primary tumors in the thoracic and lumbar spine by reviewing 9 consecutive cases treated in our institution.

## Methods and Materials

### Patients

**Figure 1:**
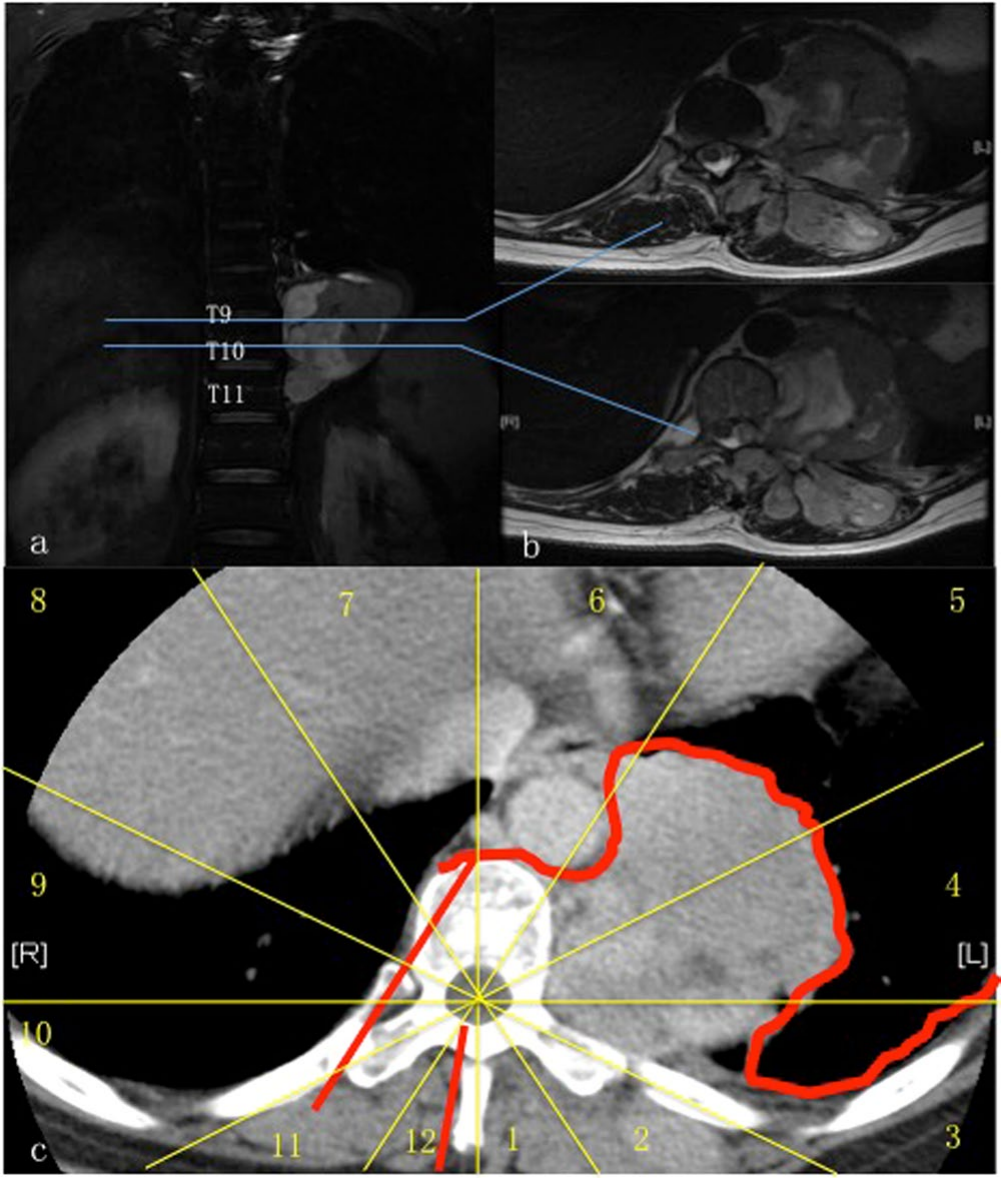
A case of a 63-year-old female with solitary fibroma at T9–11. The patient complained of numbness in the back and the abdomen 7 years after undergoing a piecemeal resection of a primary tumor in the paravertebral region of the lower thoracic spine in another hospital. Postoperative pathological diagnosis after initial surgery was solitary fibroma. **a-c**. MR images (**a-b**) and CT scans (**c**) show tumor recurrence along the left side of T9 to T11 vertebra (zone 1–5, A-D, WBB). Sagittal en bloc resection was planned based on WBB classification along the margin as highlighted in red.

**Figure 2:**
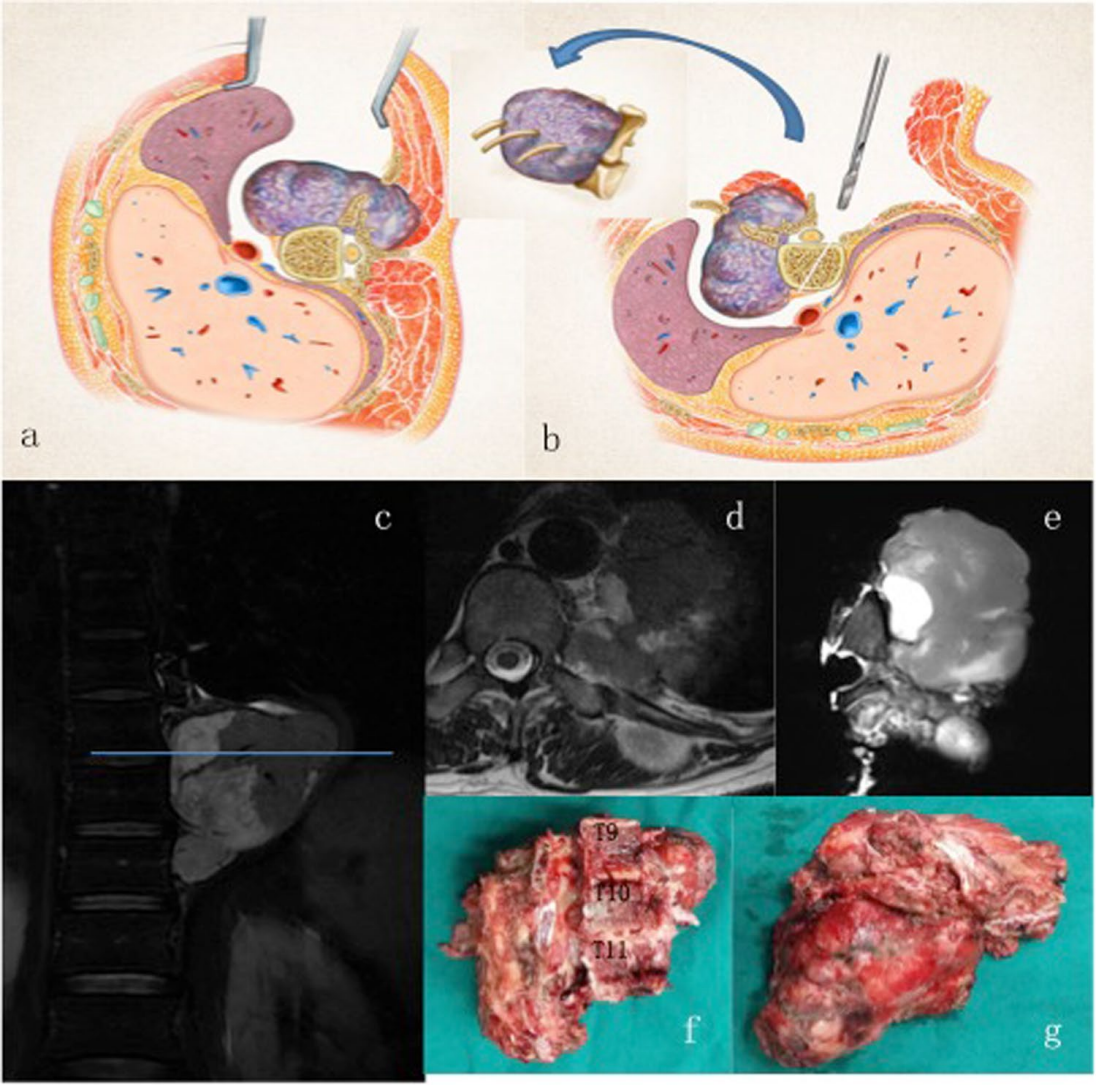
(**a**,**b**) The patient underwent a two-stage surgery in the order of anterior release (**a**) and posterior sagittal en bloc resection (**b**) with instrumentation. (**c**,**d**) A MRI transection scan of the lesion illustrates the tumor before surgery. (**e**–**g**) MR images (**e**) and photos (**f**,**g**) of the gross specimen demonstrate the margin of sagittal en bloc resection.

**Figure 3:**
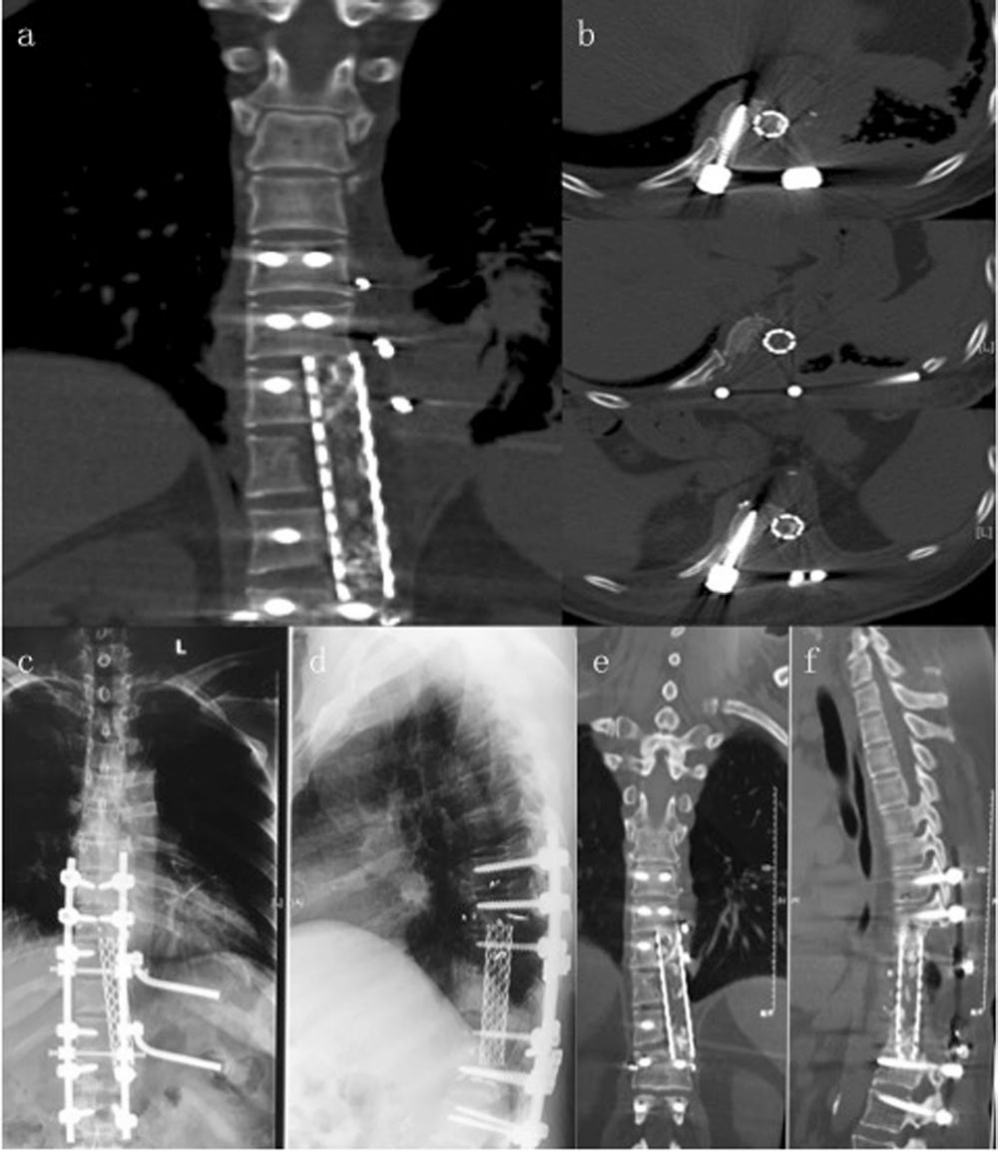
(**a**,**b**) Postoperative CT scans show structural reconstruction with instrumentation after sagittal resection. (**c**–**f**) Radiographs (**c**,**d**) and CT scans (**e**,**f**) taken at 2 years after surgery show no signs of hardware failure or tumor recurrence.

**Figure 4:**
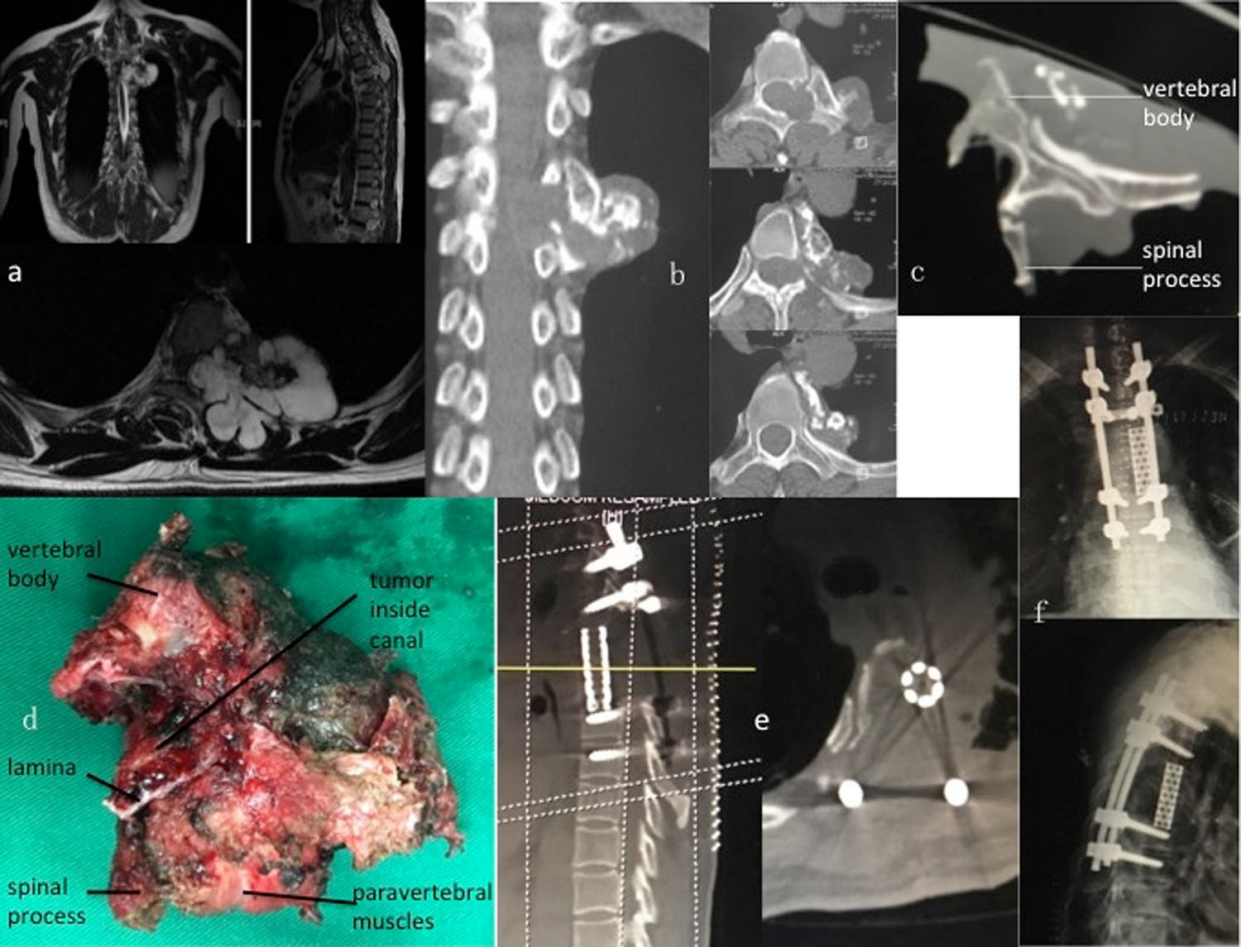
A case of 47-year-old female with chondrosarcoma at T4–5. The patient was initially diagnosed with osteochondroma after presenting with sporadic back pain for 6 years and treated with a piecemeal removal surgery 4 years before being admitted to our hospital for recurrent back pain. Postoperative pathological diagnosis after initial surgery was osteochondroma. Biopsy in our hospital suggested a diagnosis of chondrosarcoma. (**a**,**b**) Preoperative MR images (**a**) and CT scans (**b**) show tumor invading the left side of T4 and T5 vertebra (zone 1–6, A–D, WBB). The patient underwent a two-stage surgery in the order of anterior release and posterior sagittal en bloc resection with instrumentation. She experienced a delayed cord ischemia since 20 hours after the second procedure, with Frankel C cord damage at the worst, and recovered spontaneously within two weeks. (**c**,**d**) A CT scan (**c**) and a photo of the gross specimen (**d**) illustrate the margin of sagittal en bloc resection. (**e**,**f**) Postoperative CT scans (**e**) and radiographs (**f**) show structural reconstruction with implantation of a titanium mesh. Follow-up at 20 months after surgery showed no signs of hardware failure or tumor recurrence.

This study involved in 9 consecutive patients with primary tumors in the thoracic and/or the lumbar spine treated in our hospital during 2016 to 2018 (Table 1). There were 8 females and 1 male, with a mean age of 35.9 years (14–63 years). Types of tumor included chondrosarcoma (5 cases), leiomyosarcoma (1 case), giant cell tumor (1 case), solitary fibroma (1 case) and osteoblastoma (1 case). Enneking classification grades were S3 (3 cases), IB (4 cases) and IIB (2 cases). Two cases were recurrent tumors (Figs. 1–4). There were 7 cases of thoracic spine tumor and 2 cases of lumbar spine tumor. Tumors involved one level in 1 patient, two levels in 5 patients and three levels in 3 patients. In all cases, tumors were within either zone 1 to 6 or zone 7 to 11 and between layer A to D according to the WBB staging system. All patients were free of neurological deficits before surgery.

**Table 1:** Patient information.

Age	Sex	Tumor	Level	Enneking	WBB	Intra-op complications	Post-op complications	Follow-up (mons)
63	F	solitary fibroma	T9–11	S3	1–5,A-D	no	skin flap necrosis	24
35	F	chondrosarcoma	L2–3	IB	1–4,A-D	no	L2 root injury	24
47	F	chondrosarcoma	T4–5	IB	1–6,A-D	no	delayed cord ischemia	20
33	F	osteoblastoma	T8–10	S3	1–4,A-D	cord injury	no	27
38	M	chondrosarcoma	L4	IB	1–4,A-B	iliac vein rapture	no	27
14	F	chondrosarcoma	T2–3	IIB	1–5,A-C	dura tear	no	13
17	F	leiomyosarcoma	T11–12	IB	1–5,A-C	no	no	16
40	F	giant cell tumor	T4–6	S3	7–11,A-C	no	no	14
24	F	chondrosarcoma	T8–9	IIB	8–12,A-B	no	no	19

### Treatment decision making

All diagnosis was made based on histopathology reports of core needle biopsy. CT, MRI, radiograph of the spine and positron emission tomography were performed on all patients. Strategy of treatment for each patient was determined by the same multidisciplinary team of surgeons, pathologists, radiologists, radiotherapists and chemotherapists. Each time after the surgical plan was made, the patient was thoroughly briefed regarding the procedure and the morbidity associated with it. Preoperative embolization was recommended in all cases but not performed in 3 for financial reason. All surgical operations were performed by the same surgeon.

### Surgical procedure

Sagittal resection was planned for each patient based on WBB classification with consideration of surrounding neurovascular structures as identified by radiographic studies. All patients underwent combined procedures in the order of anterior release of tumor from surrounding neurovascular structures, posterior en bloc resection by osteotomy, instrumentation and reconstruction of the anterior defect. During release surgery, the tumor was separated from the great vessels and surrounding structures with blunt dissection all along the anterior margin. A gauze sponge was placed in separation and protection of the vessels and structures, which would later be removed in the posterior procedure. The patient was then positioned prone for posterior en bloc resection.

The posterior procedure included insertion of pedicle screws, piecemeal excision of the laminae and the pedicle that were not infiltrated by the tumor, unilateral costotranversectomy (in cases of ribs involvement), ligation of the nerve roots (in cases of infiltration), and release of the dura from the tumor. The caudad and cephalad discs were severed using a bone scalpel before a temporary rod was fixed on one side. Malleable retractors were placed anterior to the vertebral bodies in order to protect the surrounding neurovascular structures. Osteotomy was performed from the healthy side of the spine posteroanteriorly along the sagittal plan of the vertebra(e) using an ultrasonic scalpel. Care was taken not to violate the margin of the tumor during release and resection procedure. The tumor, along with all infiltrated structures on the affected side (nerve roots, dura etc), was then removed from posteriorly using a rotating maneuver. Each removed sample was sent for CT scans and histopathologic analysis to investigate the resection margin. The caudad and cephalad end plates were then curetted to prepare vascular beds for fusion. The gauze left from the release procedure was removed.

Posterior instrumentation including pedicle screws and rods (Wego, Weihai, Shandong, China) fixation was extended to 2 levels above and below when no more than 2 vertebrae were resected, and to 3 levels when more vertebrae were resected. Anterior reconstruction was carried out using a titanium mesh (Wego, Weihai, Shandong, China) filled with rib autograft (2/6) or allograft (4/6) in 6 cases, or a customized 3D printed artificial vertebra in 2 cases. In one case, no interbody implant was needed as less than a quarter of the vertebra was removed for en bloc resection.

### Post-op care

All patients were transferred to intensive care unit (ICU) immediately after surgery for safety consideration. Surgical drainage was kept until the volume was less than 100 ml. Patients were mobilized with the protection of brace as soon as the drainage was removed. Further adjuvant radiotherapy or chemotherapy was carried out based on post-operative histopathologic results.

### Follow-up study

Radiographs, CT scans and MR imaging were taken of each patient before hospital discharge and during follow-up, monitoring the sites for residual lesions, recurrence and hardware failure, as well as signs of metastasis. Interbody fusion was defined as the presence of trabecular bridging bone formation between the involved vertebral bodies in or out of the interbody implant as shown by radiographs and/or CT scans.

### Ethical approval

All procedures performed in studies involving human participants were in accordance with the ethical standards of the institutional and/or national research committee (Peking University Third Hospital Medical Science Research Ethics Committee IRB00006761) and with the 1964 Helsinki declaration and its later amendments or comparable ethical standards.

### Accordance

All methods were in accordance with the relevant guidelines and regulations. The experimental protocol was approved by Peking University Third Hospital Medical Science Research Ethics Committee, IRB00006761.

### Informed consent

Informed consent was obtained from all individual participants included in the study.

## Results

In 8 out of 9 cases, anterior approach was preformed for tumor release before posterior en bloc resection by sagittal osteotomy, removal of tumor and instrumentation. One patient with a L4 chondrosarcoma underwent an anterior-posterior approach for tumor release and osteotomy with an additional lateral approach for tumor removal in order to avoid damaging the nerve roots. Six patients went through all procedures at the same day; three patients underwent the second procedure at a different date for safety concerns raised by prolonged surgical time.

En bloc resection was achieved in all patients. Postoperative CT scans and histopathologic analysis indicated that wide resection margin was achieved in 7 patients (78%), marginal and intralesional margin was achieved in 1 patient (11%) each. In both these latter 2 cases, tumors were in contact with the dura. One patient had the tumor removed without breach of the capsule, while the other did not and subsequently underwent postoperative radiotherapy.

The mean operating time and blood loss were 613 minutes (241–1002 minutes) and 1867 ml (500–5250 ml). Two patients with lumbar spine tumors had more intraoperative bleeding (3000–5250 ml) than the rest seven patients with tumors in the thoracic regions (500–2450 ml). There were 4 incidences of major complications (44%) and 2 minor complications (23%) in 6 patients, with an overall perioperative complication rate of 67%. A major complication was defined as one that substantially alters an otherwise full and expected course of recovery; others were defined as minor^8^. Intraoperative major complications included massive bleeding due to rapture of the iliac vein in one patient who fully recovered afterwards, and spinal cord injury in another who was left with Frankel C cord damage instantly and Frankel D damage at 27-month follow-up. The other 2 major complications occurred in 2 patients respectively, within 3 weeks after surgery. One experienced delayed ischemia of spinal cord but fully recovered in two weeks; another had necrosis of skin flap at the wound that required additional surgery to close. The 2 minor complications, including nerve roots injury (1 case) and dura tear (1 case), both occurred during surgery. In both cases, patients fully recovered during the follow-up.

The mean follow-up period was 20.4 months (13–27 months). All patients were alive, free of neurological deficits and mobile without assistance except for the one with spinal cord injury who was walking with a crutch at the last follow-up (Frankel D). There were no evidences of residual lesion, recurrence, metastasis, or hardware failure found by using radiography, CT scan and MR imaging. Interbody fusion was confirmed with CT scans in 4 (2 with autograft, 2 with allograft) of the 6 patients (67%) who were implanted with interspace titanium meshes. Fusion was not assessable in patients with 3D printed artificial vertebrae as the implants were largely radiopaque. Normal spinal alignment was maintained in all patients (9/9).

**Figure 5:**
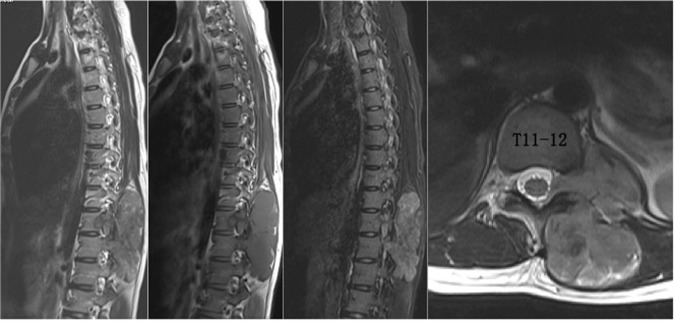
A case of a 17-year-old female with leiomyosarcoma at T11–12. The patient who complained of feeling a protruding mass in the back was found with a lesion in the thoracic spine on MRI scans. MR images show that the tumor involves the left side of T11–12 vertebra (zone 1–5, A–C, WBB).

**Figure 6:**
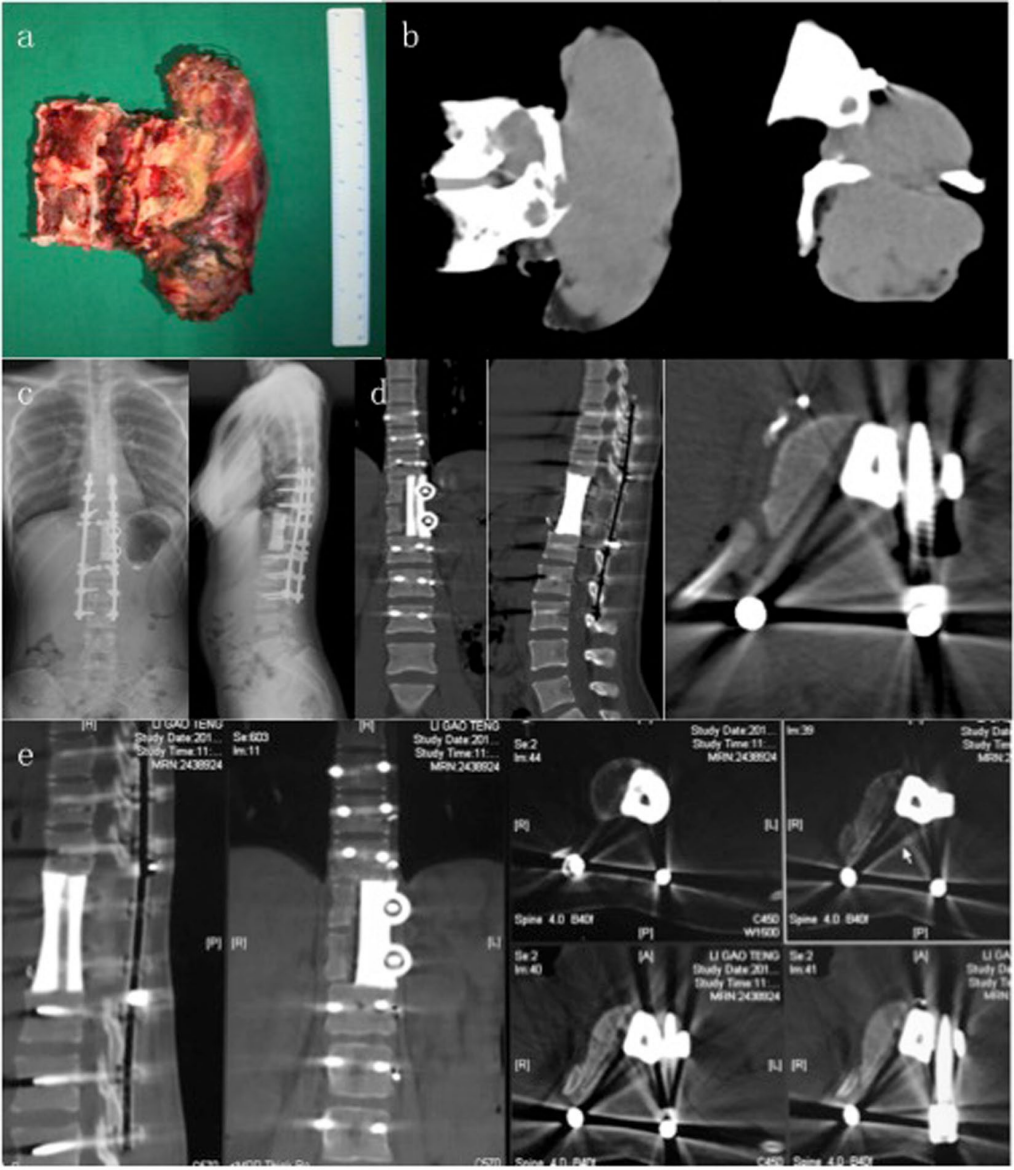
The patient underwent one-stage combined anterior release and posterior sagittal en bloc resection with instrumentation. (**a**,**b**) A photo (**a**) and a CT scan (**b**) of the specimen removed by sagittal en bloc resection. (**c**,**d**) Postoperative radiographs (**c**) and CT scans (**d**) illustrate structural reconstruction with implantation of a customized 3D printed artificial vertebra. (**e**) CT scans taken at 1 year after surgery show no signs of instrument failure or tumor recurrence.

**Figure 7:**
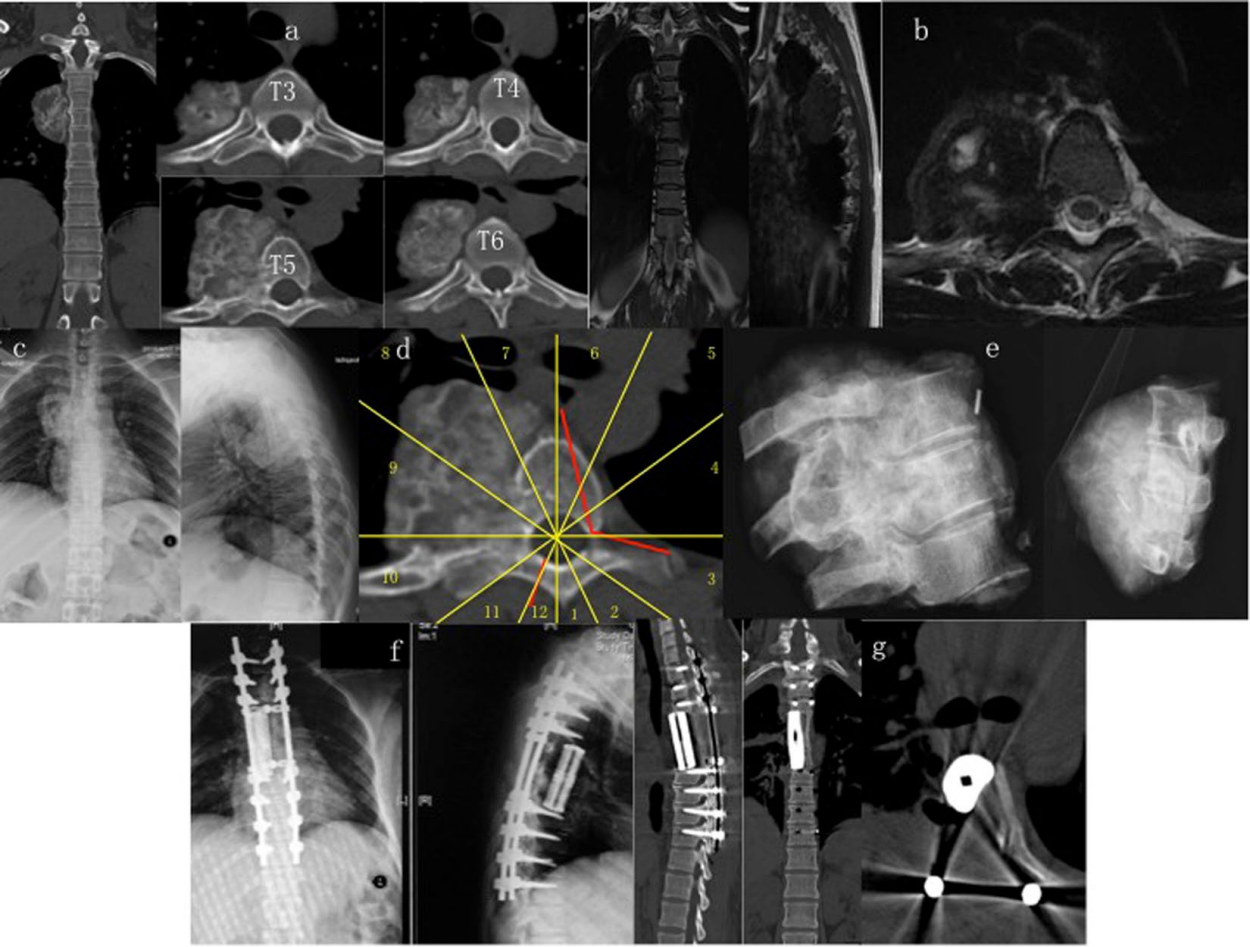
A case of 40-year-old female with giant cell tumor at T4–6. The patient who had presented with back pain for 3 months was found with a lesion in the thoracic spine on CT scans. (**a**–**c**) CT scans (**a**), MR images (**b**) and radiographs (**c**) show tumor developing on the right side of T4–6 vertebra (zone 7–11, A–C, WBB). (**d**) Sagittal en bloc resection was planned based on WBB classification along the margin as highlighted in red. She underwent a one-stage combined surgery in the order of anterior release and posterior sagittal en bloc resection with instrumentation. (**e**) Radiographs of the specimen removed by sagittal en bloc resection. (**f**,**g**) Postoperative radiographs (**f**) and CT scans (**g**) illustrate structural reconstruction with implantation of a customized 3D printed artificial vertebra.

Figures 1–7 illustrate the process of sagittal en bloc resection in 4 of our patients.

## Discussion

Sagittal en bloc resection for primary tumors in the thoracic and lumbar spine was proposed by Boriani *et al*. based on WBB classification^7,9^. It is indicated for tumors that originate and develop eccentrically in the vertebral body, the pedicle, the transversal process, or the paravertebral region (zone 2–5 or 7–11). The proposed approaches are either posterior approach alone or combined anterior and posterior approach for release and en bloc removal of the tumor. En bloc resection involves in a sagittal osteotomy via the healthy half of vertebra(e) with a safe distance from the margin of the tumor. The aim is to remove the tumor with a safe margin while avoiding excessive dissection of surrounding tissues and preserving as much integrity of the spinal column as possible. Although the proposition sounds promising, a systematic review with search of Medline and Pubmed MeSH has revealed no more than sporadic case reports concerning the use of sagittal en bloc resection for primary spinal tumors in the thoracic and lumbar region, likely due to its specific but limited indications. A study with a larger sample size like ours, presents a valuable opportunity for further investigation on this issue. Our study aims to investigate this procedure by answering the following questions: 1) Can it remove the tumor with a safe margin? 2) Would it result in adequate survival rates and local control in the tumors? 3) What are the complications associated with it? 4) Can stability be restored after resection of tumor with acceptable function?

All of our patients had paravertebral tumors located within zone 1–6 or 7–11 and hence met the indication of sagittal en bloc resection. The advantages of a combined procedure in the order of anterior release and posterior resection are that it provides direct visualization to the ventral structures of the spinal column, which reduces the risk of damaging the vessels and the safe margin of resection, and allows posterior reconstruction to be implemented while the patient was in a prone position^10^. All combined procedures were performed simultaneously whenever it was safe in order to reduce ICU stays.

This procedure achieved wide and marginal resection in 89% of our patients as confirmed by postoperative imaging and histopathologic analysis. Accordingly, it was associated with a 100% 1-year survival rate and local control rate. Similar procedure of anterior-posterior approach for complete removal of thoracic paravertebral dumbbell tumor has been reported before with successful results^11–13^. Cappuccio *et al*. described their experience in thoracoscopy-assisted sagittal en bloc resection of paravertebral tumors in 2 patients, including colon cancer metastasis at T11 and an osteosarcoma at T5–6. They also used combined procedure of anterior release followed by posterior osteotomy with instrumentation. Both patients were disease free at 2-year follow-up. These high percentages of negative margin and local control achieve by sagittal en bloc resection are comparable to the rates reported in combined anterior-posterior TES (71–100% and 90–100%)^14–16^. All surgical margins in this study were planned strictly based on WBB classification, which had been verified as a highly valid staging system capable of accurately predicting the margins in most of the cases (73–75.7%)^17,18^. Successful prognosis is to be expected, as negative surgical margins provided by en bloc resection are the key factors affecting the oncologic outcome in aggressive benign and malignant tumors^17^.

The mean operating time and blood loss, in this study, were 613 minutes and 1867 ml. Comparison of these figures in the literature is hardly precise, given the heterogeneity in different studies. But one of the largest reviews, including 229 patients with spine tumors, associated TES with 12.1-hour average operating time and 3700 ml average blood loss^10^. Tomita *et al*. reported their 14 years’ experience of TES in 98 cases of spine tumors^19^. At the end of the learning curve, they managed to reduce average operating time and blood loss of posterior TES, for a single segment in the thoracic region, to 6–8 hours and 1300 ml. Several similar studies of combined anterior-posterior approach TES in the thoracic and lumbar region reported the corresponding figures in the range of 6.8–18 hours and 3200–4000 ml^17,20,21^. Our results of comparatively shorter operating time and less blood loss are not surprising as sagittal resection involves in only half of the vertebral column with less dissection and manipulation of blood vessels in comparison with TES.

The morbidity of en bloc resection of spinal tumors is considerable because of the manipulation of neurovascular structures required in these procedures^5^. Our overall perioperative complication rate (67%), including major (44%) and minor complications (23%), is in line with that of combined anterior-posterior TES as reported in the literature (46% to 65%)^6,14–16,22^. Fourney *et al*. treated 26 patients who had thoracic and lumbar spine tumors with combined anterior-posterior radical resection and reported 9 cases of major complications (35%) and 5 cases of minor complications (19%)^16^. One possible explanation of the slight higher major complication rate in our study is that, in our series, 8 patients underwent multilevel resection and 2 patients had recurrent tumors after previous surgery. Large series studies on TES have associated multilevel resection and revision surgery with significant increase in major complication rates^23^. Major complications in our study include iliac vein rapture (1/9), spinal cord injury (1/9), nerve roots injury (1/9) and skin flap necrosis (1/9), which are also among the most common complications in TES^9^. Damage to ventral vessels of the vertebral column is commonly seen in all TES procedures in the thoracic and lumbar region, particularly one-stage posterior approaches, due to a lack of visualization of the anterior structures^10,24^. This risk is significantly reduced in a combined anterior-posterior approach as it provides direction visualization of the anterior vertebral column. Manipulation or sacrifice of vascular and nervous structures are also among the common causes of morbidities of TES^24^, which can be reduced if surgical disturbance is limited to one side of the vertebral column, as in a sagittal en bloc resection procedure. We performed the combined procedure simultaneously in 6/9 of our cases, whereas evidences have suggested that the risk of complications in en bloc resection with combined approaches can be reduced if the procedure is staged at separate time^8,9^. Noticeably, no patient died in our study; whereas a literature review suggests that mortality rate of TES in the thoracic and lumbar region is in the range of 0 to 7.7%^17,21,24^. Presence of these complications demonstrates the risk of the sagittal resection procedure, but it is reasonable to presume that the risk would be higher, should more extensive dissection as in TES is carried out on the same patients.

Sacrifice of bones, muscles, ligaments and anatomical barriers required in en bloc resection leads to spinal instability that demands complex circumferential reconstruction^16,25^. The standard reconstructive technique involves in posterior pedicle screws, rods and anterior interspace constructs filled with autogenous graft or bone substitutes^26^. In our study, interspace titanium meshes filled with autogenous or allogenic graft were used in 6 patients, resulting in a 67% fusion rate. Two patients were implanted with 3D printed artificial vertebrae because these devices were designed to further promote spinal fusion with superior surface and patient-specific anatomical shape^27,28^. Although interbody fusion was not confirmed in all of our patients, none of them was found with evidences of hardware failure. Restoration of stability was also reflected in that all but one patient with spinal cord injury were fully mobile at the last follow-up. Smitherman *et al*. and Oppenlander *et al*. published 2 individual case reports of multiple segments anterior-posterior sagittal en bloc resection of a giant cell tumor and a chordoma in the thoracic spine^29,30^. They both used allograft mixed with bone substitutes as spacer, instead of interspace implants. Both patients experienced no hardware failure at the last follow-up (1 year and 6 months respectively). All these results of sagittal resection appear to be better in comparison with the 2.8% to 20% hardware failure rate reported in combined anterior-posterior TES^15,16,20,22,31^. This can be explained as diminished tissue vitality from extensive intraoperative vascular dissection in TES can weaken the instrumentation with a poor healing capacity^15,22^. Moreover, hardware would be less burdened if more of the integrity of the spine were to be preserved^19^.

One limitation of this study is the relatively small sample size. However, it is larger than any other similar studies published in the literature. As the indications for this surgery are limited, so will be the sample size. Further studies with control groups are required to establish a definitive role of sagittal en bloc resection in the treatment of spinal tumors, but considering the rarity of these conditions, such data will be hard if not impossible to come by. This study could also be criticized for its heterogeneity in terms of the types of tumors involved and short follow-up time. But it is the technical procedure, rather than the biological behavior of the tumor that is of interest to this study. Resection with a safe margin is the safeguard of long-term result. Given that the time criterion for follow-up in the study of spine tumor is largely subjective, it varies in the literature. More than half (5/9) of our cases were chondrosarcoma, for which a 2-year follow-up time was adopted in one of the largest systematic review^32^. Our results, with an average 21-month follow-up, err on the side of caution.

## Conclusion

Combined anterior-posterior sagittal resection based on WBB classification is a feasible, safe and effective procedure for en bloc resection of paravertebral primary tumors involving the thoracic and lumbar spine. The procedure is reliable in achieving safe oncologic margins with promising survival rates and local control. The morbidity and high complication rate associated with this procedure should not deter an experienced surgeon from performing this surgery when indicated, as patients tend to recover from their complications in majority of the cases. Loss of stability caused by surgery can be restored by using conventional devices as well as 3D printed implants with favorable function and minimal implant failures. Although the surgical procedure can be both mentally and technically challenging, our encouraging results favor and validate this technique.

## Data Availability

The datasets generated and/or analyzed during the current study are available from the corresponding author on reasonable request.
